# Reciprocal Inuit and Western research training: facilitating research capacity and community agency in Arctic research partnerships

**DOI:** 10.1080/22423982.2018.1425581

**Published:** 2018-01-31

**Authors:** Priscilla Ferrazzi, Peter Christie, Djenana Jalovcic, Shirley Tagalik, Alanna Grogan

**Affiliations:** ^a^ Faculty of Rehabilitation Medicine, Department of Occupational Therapy, University of Alberta, Edmonton, Canada; ^b^ Science Communication, Edmonton, Canada; ^c^ Aqqiumavvik Society, Arviat, Nunavut; ^d^ Political Science and History, McGill University, Montreal, Canada

**Keywords:** Research training, Indigenous and Western ethics, Inuit and Western ethics, Inuit Qaujimajatuqangit, research methods, community agency, research capacity, research partnerships, Arctic

## Abstract

Engaging community partners to work as co-researchers and research assistants for research involving Inuit communities or regions helps to ensure the equitable recognition of community and researcher priorities, mutual trust and respect, participation by local participants, inclusion of local knowledge and local uptake of research findings. However, research knowledge still in development among community members has been described as a barrier to effective Arctic community research partnerships. This paper describes two 3-day, cross-cultural research training workshops held in the Nunavut communities of Arviat and Iqaluit during Spring 2017. The purpose was to encourage reciprocity as a basis for research training that incorporates both Western and Inuit approaches and that emphasises relationship building to benefit both Inuit and non-Inuit research communities. A review of participant responses to the workshops suggests value in using an integrated Western–Inuit framework of educational objectives to guide the training. Responses suggest the workshops helped improve understanding of research practices and ethics rooted in different traditions for participants interested in assisting with or conducting research in Canada’s Arctic communities.

## Introduction

Canadian Arctic research partnerships involving university researchers and Inuit communities are increasingly seen as essential to an improved understanding of the region by Arctic residents and by academics [1]. As the number of research projects in Inuit communities continues to rise [2], partnerships between these communities and academic researchers are becoming more collaborative and responsive to local priorities [3]. The need for Indigenous community involvement is explicitly addressed in the research licensing requirements in the 3 Canadian territories [3] and by Canada’s federal research granting agencies through the *Tri-Council Policy Statement: Ethical Conduct for Research Involving Humans* (TCPS2) [4], although many institutional barriers to such involvement persist. Inuit communities are calling for participation at every research level for studies taking place in their regions [2,3]. While the Canadian International Polar Year research programme was an important step towards meeting this call with scientists, communities and Inuit and other Indigenous leaders working together on northern projects [5], its impact could have been more significant had there been a cohort of community-based trained research assistants available for deeper partnerships. For many Indigenous people, control of the research agenda within their territory is a recent movement considered essential to Indigenous governance, self-determination and identity [6–8]. Early community–researcher engagement is also widely perceived as an important factor affecting positive research outcomes [9], including the inclusion of local knowledge and local uptake of research results [7], and for building trust between Inuit communities and researchers [4,8].

Nevertheless, challenges exist for ensuring Arctic research equitably involves and benefits both local communities and academic researchers from elsewhere [5,9]. Many say “colonial research approaches” continue to be practised in the North [7, p.102], and communities remain generally perceived to receive less benefit from research than their academic counterparts [9]. Research in which Indigenous people are excluded from research participation while remaining subjects of study often fails to positively affect the communities involved, while risking harm through the perpetuation of colonial values [10]. Canadian health research, in particular, has historically not considered Indigenous peoples and communities as “primary stakeholders of research evidence” [11]. Consequently, many Indigenous communities in Canada view research and researchers with apprehension and mistrust [4]. Among the consequences of this mistrust can be low recruitment of study participants who see no benefit from the research to them or their community or fear the risk of stigma, mistreatment or exploitation [12]. Although many Inuit recognise the value of research on their land and in their communities [13], trust appears an essential prerequisite to research success where Inuit communities are involved [8,11].

One widely acknowledged approach for encouraging trust and more equitable community–researcher partnerships in Inuit communities is the facilitation of employment among local researchers and research assistants [9,14]. Local personnel can bring considerable value to research projects given locals’ competencies and mastery of their own social, geographic and cultural environment. Their familiarity with the local context can improve communication between communities and researchers [15], facilitating culturally appropriate strategies for recruitment [16], data gathering [17], interpretation and verification of data [14]. Employment of local researchers can lead to a mutually beneficial and authentic reciprocal relationship between communities and academic researchers. Ultimately, the biggest challenge to successful research partnership has been the accountability of academic researchers to bring findings back to the community in a meaningful way. For community, the point of partnership is to gain knowledge and insights that can positively impact community concerns. The employment of local research assistants helps to ensure that the entire research process more fully engages communities.

Developing ways of effectively reporting back to communities and research knowledge that is still to be developed among community members interested in research have been described as barriers to effective community research partnerships [8]. Indeed, a lack of local involvement, including involvement of community researchers, is considered among the chief concerns of Inuit communities regarding research partnerships [13]. Cross-cultural research training for Inuit students and community members along with non-Inuit university students and researchers is one approach to answering these concerns. Research training in communities involved in research partnerships can encourage dialogue, advance research and foster collaboration [18]. In many cases, research training can be considered a metric of research success in itself. For example, in their recent survey of research-involved community members and researchers, Brunet and colleagues [9] found that respondents considered “training, new skills and professional growth for students and engaged locals” to be the most important perceived positive outcome of research partnerships. This finding was followed closely by “motivation, inspiration and empowerment for local partners”. Similarly, training or activities for researchers unfamiliar with Inuit regions or communities can improve cultural competencies and a shared understanding of research ethics that increase the likelihood of research success [2].

This paper describes two 3-day cross-cultural research training workshops, entitled *Reciprocal Inuit/Graduate Student Research Assistant Training in the Canadian Arctic*, held in the Nunavut communities of Arviat and Iqaluit in the spring of 2017 [19,20]. The workshops were designed as a cross-cultural collaborative project of the Aqqiumavvik Society in Arviat and the University of Alberta (Faculty of Rehabilitation Medicine, the Health Sciences Education and Research Commons, and Library Services). The purpose was to encourage reciprocity as a basis for research training and relationship building that benefits both Indigenous and non-Indigenous research communities. A learner-centred approach was used to build skills needed to conduct research and to highlight culturally responsive research methods, advancing an agenda of building capacity for conceptual understanding of culturally responsive ethics in research. The workshops were conducted in the context of training research assistants from Nunavut communities and the University of Alberta and elsewhere to participate in a 2-year, interdisciplinary health-and-law research project in Canada’s Arctic [21,22]. A review of participant responses following the workshops explored the usefulness of the workshops from participant perspectives and, in particular, the effectiveness of integrating a common, internationally recognised framework of educational objectives (Revised Bloom’s Taxonomy) [23] with research knowledge and educational concepts from Inuit knowledge (*Inuit Qaujimajatuqangit* or IQ) to achieve cross-cultural workshop goals.

## Method

Research training workshops were held over 3 days in each of the Nunavut communities of Arviat (pop. estimate: Inuit pop. 2,591; non-Inuit pop. 181) and the territorial capital of Iqaluit (pop. estimate: Inuit pop. 4,208; non-Inuit 3,382) [24] during April and May 2017, respectively. The workshops featured presentations by Arctic researchers, Inuit Elders (Arviat only), Inuit research assistants, graduate students and others. Select workshop sessions were delivered using videoconferencing technology linking the University of Alberta and other universities to the Iqaluit research training site.

### Recruitment

Workshop participants were purposively recruited following guidance from the Nunavut collaborator and using word-of-mouth, a government public service announcement, a presentation at the Arviat high school and an advertisement on the Nunavut Research Institute website. While the project sought to recruit research assistants, the workshops were made more widely available to those interested in research. Participants included Inuit community members interested in research, current Inuit and non-Inuit research assistants, undergraduate students, Arctic College students and high school students in Arviat and Iqaluit. Thirty-five participants attended (Inuit and non-Inuit), n = 18 in Arviat and n = 17 in Iqaluit. Participant retention for the full 3 days of the workshop was 100% in Iqaluit with attendance more fluid in Arviat, given commitments of high school students. Attendance was not remunerated except 5 participants identified in advance of the workshops as interested in working as research assistants on the interdisciplinary health-and-law project who were salaried. (Arviat workshop participants were also provided 1 h of research assistant wages for working on a “real-life” research task relevant to the health-and-law research project.)

### Workshop framework and programmes

The workshop framework (Table 1) was derived from a modification of the Revised Bloom’s Taxonomy of educational objectives [23] with taxonomy competencies integrated with IQ philosophy through (a) holistic application, (b) engaging learners at various learning stages and (c) collaborative learning (i.e. *piligarinaq* or “working together for common purpose”). In particular, the modified framework allowed for a holistic approach spanning multiple competencies and engaging participants from all stages of learning, while setting realistic learning objectives. The open, collaborative forum encouraged participation and validated the relevance of lived experience through respectful and inclusive sharing in group work and related presentations as well as a round-table in Iqaluit. Discussions emerging specifically from the analysis of scenarios allowed the participants to work through situations to which they could relate directly from personal knowledge. The workshops engaged learners from all 5 stages of demonstrated skills and abilities reflected in the Nunavut IQ Education Framework (Supplementary Table 1) [25]: (1) *qaujilisaaqtuq*: the emergent learner (listening and observing); (2) *tukisiliqtuq*: the transitional learner (using information and skills); (3) *tukisinaqsiliqtuq*: the communicative learner (growing in confidence, in resourcefulness and with open communication); (4) *pinasugunnaqsijuq*: the confident learner (applying new knowledge and engaging in dialogue and collaboration); and (5) *pijunnaqsijuq*: the proficient learner (interpreting, deepening meaning and understanding).

**Table 1.** Learning modules (based on a modified Revised Bloom’s Taxonomy).

Module	Module 1 Understanding Research, Context and Traditional Knowledge	Module 2 Negotiating Relationships with Communities and Research Ethics	Module 3 Research Skills
Topics	What is research?	Perceptions of research in Arctic communities	Qualitative research
	*Inuit Qaujimajatuqangit* explained	Researcher relationships with communities	Indigenous and Western ethics
	*Inuit Qaujimajatuqangit* and research	Research licensing in Nunavut	Indigenous and Western approaches to research
	Nunavut Land Claims Agreement	Inuit perspectives on research ethics	Blended approaches
	Government of Nunavut	Introduction to the Tri-Agency framework for responsible	Recruiting study participants
	Legal system and access to justice	Conduct of research; and Tri-Council Policy Statement 2 (TCPS 2 – Chapter 9 Research Involving the First Nations, Inuit and Métis Peoples of Canada)	Effective interviewing skills
		Consent	Interviewing and cultural considerations
		Confidentiality	
Approach	*Inuit Qaujimajatuqangit* (IQ), Elder presentations (Arviat), Participant IQ round-table (Iqaluit), presentations, group work and reflection	Panel, presentations, video-link presentation (Iqaluit), group discussion, skills practice and reflection	Presentations, video-link presentation (Iqaluit), group discussion, skills practice and reflection
Stages in Learning Continuum (Inuit Qaujimajatuqangit Education Framework)	Emergent, transitional and confident	Emergent and transitional	Emergent, transitional, communicative, confident and proficient
Knowledge Dimension (Revised Bloom’s Taxonomy)	Factual and conceptual	Conceptual and procedural	Factual, conceptual and procedural
Cognitive Process (Revised Bloom’s Taxonomy)	Understand	Understand	Understand, apply and analyse

Recognising the short duration of the workshops, the main knowledge dimensions explored were conceptual and procedural (Table 2). Conceptual knowledge is about interrelationships among elements that enable them to work together within a larger framework while procedural knowledge is about how to do something and includes methods of inquiry and skill-based knowledge [23]. Conceptual knowledge in this workshop series related to principles of IQ as well as Inuit-and-Western worldviews, ethical frameworks and legal notions. Procedural knowledge related to knowledge of Western and Indigenous qualitative methods of inquiry, their integration, steps in the research process and related research skills. The cognitive process dimensions used were intended to encourage participants to understand, apply and analyse the material. Details of the workshop framework, goals, learning outcomes and programmes are described by Ferrazzi, Jalovcic and Tagalik [19,20].

**Table 2.** Dimensions in the Revised Bloom’s Taxonomy (Krathwohl, 2002).

Knowledge dimensions	
Factual Knowledge	Basic elements that learners must know to be acquainted with a discipline or solve problems in it (e.g. knowledge of terminology)
Conceptual Knowledge	The interrelationships among these basic elements within a larger structure that enable them to function together (e.g. knowledge of classifications, theories and models)
Procedural Knowledge	How to do something; methods of inquiry; and criteria for using techniques and methods (e.g. Knowledge of subject-specific skills)
Cognitive Process Dimensions	
Understand	Determining the meaning of instructional messages, including oral, written and graphic communication (e.g. interpreting, classifying, summarising and explaining)
Apply	Carrying out or using a procedure in a given situation (e.g. executing and implementing)
Analyse	Breaking material into its constituent parts and detecting how the parts relate to one another and to an overall structure or purpose (e.g. differentiating, organising and attributing).

### Participant evaluations

Participants were encouraged to fill out a brief final evaluation comprising written questions with a choice of answers as well as open-ended questions at the conclusion of the workshops on the third day. An additional daily feedback form for the first 2 days was added to the Iqaluit workshop. The quantitative part of the final evaluations invited respondents to identify their level of agreement with a series of statements (on a scale of 1, “strongly disagree”, to 4, “strongly agree”) such as the usefulness of the information, how effectively material was presented and overall enjoyment of the workshop. Participants were also asked to provide qualitative responses to general questions, such as “what was the most important new piece of information you learned from this workshop?”, “what was the most important new skill that you learned in this workshop?” and “did this workshop encourage you to work as a research assistant or continue to work as a researcher?” Responses were coded, organised and analysed through conventional qualitative content analysis [26] to identify categories and themes.

## Findings

A total of 16 of 17 participants completed final evaluations in Iqaluit and 14 of 18 participants completed final evaluations in Arviat. Arviat participants who left the workshop early due to high school and other commitments as well as 1 Iqaluit participant did not complete the evaluation. The 30 participant responses show (1) a high degree of satisfaction with the research training workshop, with (2) all participants agreed that the information provided was useful or improved their understanding of research; and (3) (barring 1 exception), all participants agreed that the workshops encouraged them to work as a research assistant or researcher: “This workshop really awoke my interest in not only quantitative, but also qualitative research”, wrote 1 Iqaluit respondent. “I learned to think outside the box, appreciate and explore all forms of research”. Another Iqaluit participant commented on the impact of the workshop: “… I would like to go [to] university someday now”. In Iqaluit, ethics and/or differences between Indigenous and Western epistemological traditions emerged as the most important new information acquired by many participants in the workshop: “I learned about the IQ principles. We should consider the relationship between these principles and Western principles when conducting research”. Another wrote: “The most important new thing I learned from this workshop was the critical role that ethics play in all stages of the research process, from the actual conduct of research itself to the cultural responsiveness (e.g. Indigenous vs. Western ethics in this context)”. Meanwhile, learning basic terms about research methods and ethics prevailed as most important in Arviat with several participants indicating they learned “new language”.

Iqaluit participant responses to open-ended questions asking about the most important new information and skill learned suggest workshops assisted participants (1) in conceptual knowledge and understanding concerning Western and Inuit research ethics, consent, confidentiality and IQ principles (n = 10); (2) in procedural knowledge and understanding of methods of inquiry, research funding and licensing processes (n = 9); (3) in their conceptual knowledge and analytical capacity relating to the role of ethics in the research process and blending of Indigenous and Western research approaches (n = 4); and (4) in application of procedural knowledge as it relates to formulating research questions and interviewing (n = 6).

Responses to the above open-ended questions by most Arviat participants suggest the workshops assisted participants (1) in acquisition of factual knowledge and understanding of research terms largely relating to methods and ethics (n = 8); (2) in their conceptual knowledge and understanding of confidentiality and consent (n = 2); (3) in their procedural knowledge and understanding of steps in the research process and methods of inquiry (n = 4); and (4) in their application of procedural knowledge as it relates to simplification of research questions (n = 7). Four participants in the research training workshop subsequently worked as research assistants on a collaborative, community-based, health-and-law research project in Canada’s Arctic. Three of the 4 are referenced in the recruitment section of this paper and the other joined as a research assistant following the workshop.

## Discussion

Research collaboration with Inuit communities in Canada’s Arctic is increasingly recognised as important to improving research relevance and uptake, to capturing valuable local knowledge, to empowering local research stakeholders and to supporting community research capacity [9]. From the community perspective, this collaborative approach is not only a cultural expectation, but critical to assisting southern researchers in conducting themselves in culturally respectful and safe ways when engaging with Inuit communities. The Inuit principles of *piliriqatigiingniq* and *aajiiqatigiingniq* are expected processes to be used when addressing issues of shared consequence [27]. These expectations of southern researchers need to be understood and negotiated with community partners. There are also specific protocols regarding asking questions, acknowledging knowledge holders and, critically, ensuring that finding are used to improve the common good [28]. The cross-cultural research training workshops described here were developed to facilitate and encourage this collaboration.

### Integrated workshop framework

Workshop participants were generally positive about the workshop’s approach to integrating Inuit and Western concepts and approaches to research training and research ethics, and many acknowledged much of the information regarding the 2 traditions was new to them. Certainly, the approach was welcome. For centuries, European knowledge and ways of learning have been imposed on Canada’s Indigenous people through oppressive institutions such as residential schools since the arrival of European settlers while Indigenous knowledge and ways of learning have generally been diminished or negated altogether [29]. Today, while decolonising approaches to Indigenous education and training are increasingly recognised as a moral imperative [30], culturally responsive and integrated Indigenous instruction and assessment has also been demonstrated as effective for improving Indigenous education quality [31] and outcomes [e.g. 32]. In Canada, a number of post-secondary learning programmes adopt culturally responsive, inclusive and respectful approaches to Indigenous education and training, including – in the Far North – the Nunavut Arctic College (with 3 campuses and 24 learning centres in Nunavut’s 26 communities) and The Genesis Group of the Northern Learning Institute of Yellowknife [33]. The workshops described here were intended to meet similar integrated and culturally responsive needs – including the need for a holistic approach [34] for engaging all learner levels and for collaborative learning – using the Revised Bloom’s Taxonomy of educational objectives modified to adopt IQ learning principles. Workshop findings reflect differences in stages of learning with Arviat participants predominantly at the high school level and Iqaluit participants including current and graduated Arctic College and university-level participants as well as high school students. Given that post-secondary students from Arviat have to travel out of the community for further education and, therefore, were not available to participate, this difference was not unexpected.

The Revised Bloom’s Taxonomy of educational objectives is a framework of expected or intended student learning as a result of instruction [23] that was used to set realistic learning objectives. The revised taxonomy, incorporating both knowledge and cognitive process dimensions, is now widely used and accepted in many countries around the world [35] as well as in different cultural contexts. In Nunavut, current educational theory is used to align information from Elders for identifying the 5 stages in the Nunavut K-12 curriculum [25], and in one case, Bloom’s taxonomy is the basis for ideas to guide learning outcomes, understandings and competencies for integrating Inuit knowledge into Nunavut classrooms using a CD-ROM produced for that purpose [36]. While versions of the Bloom’s taxonomy have been criticised in Indigenous contexts for their absence of a spiritual dimension [30], the integration of IQ principles in the modified taxonomy for our reciprocal research training workshops appeared to answer the need for respect, focus on relationships, feelings of belonging, empowerment and purpose central to this spiritual dimension.

### Ethical worldviews in research

Collective agreement among members of a group, society or nation regarding its value system and the nature of reality is the essence of culture [33]. The workshop focus underscored the importance of research ethics as well as the relevance of culturally different worldviews that affect these ethical approaches [e.g. 37]. In the Canadian Arctic, the need for Inuit-specific research ethics is widely recognised [2] with a growing number of regional ethics boards, committees and guidelines providing direction [e.g. 38,39,40]. Increasingly Inuit communities are advancing IQ principles as the basis for authentic research partnership [37].

In particular, the history of colonisation and Western and colonial research approaches in Canada’s Arctic have often included harmful and unethical research practices [37]. The workshops described here were designed and intended to affirm the premise of Canada’s federal research funding agencies in the TCPS2 guidelines for Aboriginal research “that engagement with community is an integral part of ethical research involving Aboriginal peoples” [4, p.111]. The workshops also sought to address concerns identified earlier by the National Inuit Committee on Ethics and Research [41], including respect for language and traditional knowledge, community empowerment, a focus on positive impacts for communities and regions, knowledge sharing between researchers and Inuit communities, regions and people and clarity about Inuit roles in research [2]. Indigenous ethical frameworks do not present the same rule-based ideas of morality that Western ethical frameworks do. They are relational (based more on relationships than on the individual) and centre around the idea that the “right” action depends on the person and the circumstances. This contextual system leads to human behaviour not being regulated by telling people what to or what not to do, but rather providing a series of ideals on how to be [42]. The implications of these ethical frameworks to research include a focus on relationship building, respect for traditional knowledge and local, community engagement [43,44]. The workshop modules described here responded directly to the Inuit Nipingit recommendations for cultural competency training for researchers and students and capacity building to support Inuit interests in conducting their own research [2].

## Conclusion

Among the most effective means for Indigenous engagement and knowledge transfer is the training and remuneration of community members as co-researchers [18]. Here, we report on reciprocal Inuit and Western research training workshops in Nunavut as a useful model to answer the need for researchers and research institutions to advance a relational approach with Indigenous communities where both academic and community priorities are recognised [11]. The model promotes “transformative participation, effectively empowering community members to go beyond the particular research project in which they are employed and applying the skills and modes of thinking to new avenues of endeavor” [18, p 575]. The responses by both Inuit and non-Inuit participants suggest the workshops described here may be an effective approach for bridging cultural and ethical gaps in understanding between researchers and communities in Canada’s mainly Inuit Arctic communities, delivering both direct and indirect benefits to both.

## Supplementary Material

Table_S1.docx

## Supplemental material

The supplemental data for this article can be accessed here
