# Supplementary material for: Reciprocal Inuit and Western research training: facilitating research capacity and community agency in Arctic research partnerships
Source: Int J Circumpolar Health. 2018 Jan 31;77(1):1425581. doi: 10.1080/22423982.2018.1425581 (PMC5795698; doi:10.1080/22423982.2018.1425581)
Supplement: Table_S1.docx [file ZICH_A_1425581_SM3688.docx]

| **The Emergent Learner** (*Qaujilisaaqtuq)* | Learners begin by listening, observing and looking for patterns while beginning to build relationships with other learners and the materials. |
| --- | --- |
| **The Transitional Learner** *(Tukisiliqtuq)* | Learners become more actively engaged and begin to use new information and skills. |
| **The Communicative Learner** *(Tukisinaqsiliqtuq)* | Learners show growing confidence, resourcefulness and reflection regarding content and concepts and become more open and  detailed in communicating and relating the information to others. |
| **The Confident Learner** *(Pinasugunnaqsijuq)* | Learners are confident and in control of their learning. They apply new knowledge to multiple contexts in order to gain deeper understanding and awareness while relying heavily on dialogue and collaborative relationships. |
| **The Proficient Learner** *(Pijunnaqsijuq)* | Learners are interpreting information and building appropriate language to use in their relationships to deepen meaning and understanding, pushing forward in their application of the material, information and skills. |

*Supplementary Table 1* IQ Stages in the Learning Continuum (Nunavut Department of Education)
